# Bacterial degradation of chlorophenols and their derivatives

**DOI:** 10.1186/1475-2859-13-31

**Published:** 2014-03-03

**Authors:** Pankaj Kumar Arora, Hanhong Bae

**Affiliations:** 1 School of Biotechnology, Yeungnam University, Gyeongsan 712-749, Republic of Korea

**Keywords:** Chlorophenol, Environmental pollutants, Bacterial degradation, Biodegradation

## Abstract

Chlorophenols (CPs) and their derivatives are persistent environmental pollutants which are used in the manufacture of dyes, drugs, pesticides and other industrial products. CPs, which include monochlorophenols, polychlorophenols, chloronitrophenols, chloroaminophenols and chloromethylphenols, are highly toxic to living beings due to their carcinogenic, mutagenic and cytotoxic properties. Several physico-chemical and biological methods have been used for removal of CPs from the environment. Bacterial degradation has been considered a cost-effective and eco-friendly method of removing CPs from the environment. Several bacteria that use CPs as their sole carbon and energy sources have been isolated and characterized. Additionally, the metabolic pathways for degradation of CPs have been studied in bacteria and the genes and enzymes involved in the degradation of various CPs have been identified and characterized. This review describes the biochemical and genetic basis of the degradation of CPs and their derivatives.

## Introduction

**Figure 1:**
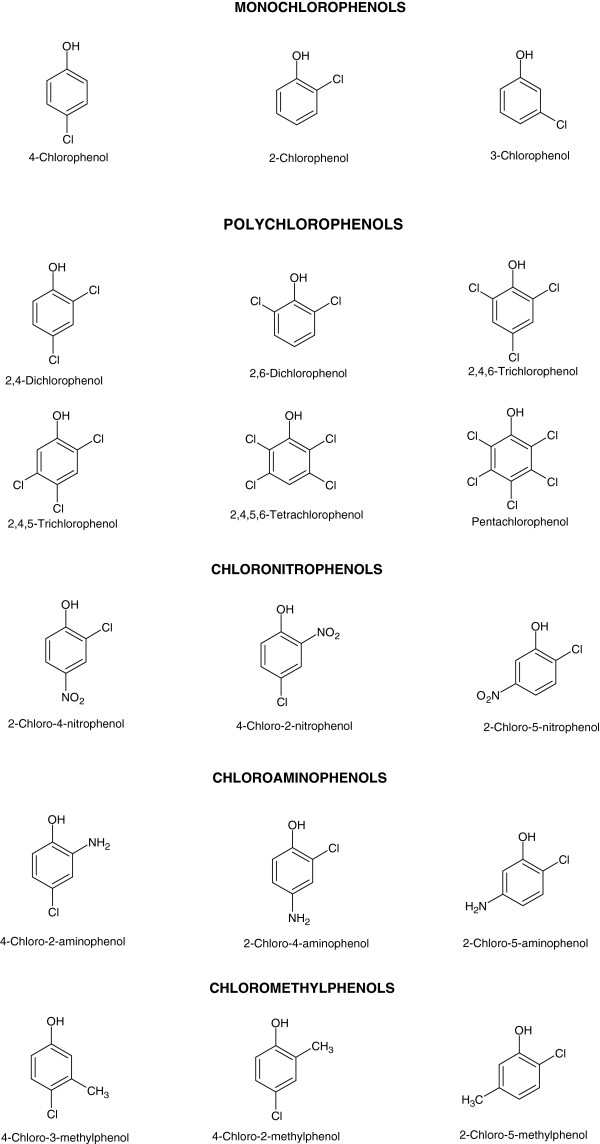
Chemical structures of chlorophenols and their derivatives.

Chlorophenols (CPs) are aromatic ring structures containing at least one chlorine atom (-Cl) and one hydroxyl (-OH) group at the benzene rings. Five groups of CPs have been recognized on the basis of their chemical structures, monochlorophenols (MCPs), polychlorophenols (poly-CPs), chloronitrophenols (CNPs), chloroaminophenols (CAPs) and chloromethylphenols (CMPs) (Figure 1). These compounds are widely used (i) as mothproofing agents, miticides, germicides, algicides, fungicides and wood preservatives [1], as well as (ii) for the synthesis of dyes and drugs [2].

CPs have been introduced into the environment via anthropogenic activities [3]. The major sources of contamination are industrial wastes, pesticides, herbicides, and complex chlorinated hydrocarbons [3]. People may be exposed to CPs by eating or drinking substances that contain them or through skin contact [4]. CPs and their derivatives are highly toxic to living beings due to their carcinogenic, mutagenic and cytotoxic properties [5]. The World Health Organization and the International Agency for Research on Cancers have characterized several poly-CPs as potential human carcinogens [5]. Similarly, the United States Environmental Protection Agency has included several CPs in its list of priority pollutants.

Several conventional methods such as adsorption, ion exchange, liquid–liquid extraction, and chemical oxidation and advanced oxidation processes have been used for the removal of CPs from wastewater [3,6]. These methods are expensive and not eco-friendly due to the formation of hazardous compounds as by-products [3]. Conversely, bioremediation is an effective and eco-friendly method of removing CPs from the environment. Biodegradation of CPs has gained attention due to the complete mineralization of CPs by microorganisms in the environment.

Several reviews dealing with the degradation and toxicity of CPs and their derivatives have been published [3,5,7,8]; however, these reviews were focused on the biodegradation/toxicity of MCPs or poly-CPs or both. The present review describes the biochemical and genetic basis of bacterial degradation of CPs and their derivatives including MCPs, poly-CPs, CAPs, CNPs and CMPs. Both aerobic and anaerobic bacterial degradation of CPs are discussed.

### Bacterial degradation of CPs

Aerobic degradation of CPs and their derivatives have been extensively investigated in bacteria, and many bacteria with the ability to utilize CPs as their sole carbon and energy sources have been isolated [8]. One of the following mechanisms may be involved in the bacterial degradation of CPs and their derivatives: (i) monooxygenases may catalyze hydroxylation at the *ortho*-positions of the chlorophenolic rings, which results in the formation of chlorocatechols that may be degraded further via *ortho-*[9] or *meta-*cleavage [10,11] or hydroxylated prior to ring cleavage [12]; (ii) monooxygenases may catalyze the hydroxylation at *para*-positions of the chlorophenolic rings, resulting in the formation of chlorohydroquinones that may be degraded further via hydroxylation [12] or dehalogenation [13] prior to ring cleavage; (iii) the degradation of CNPs may be initiated via hydroxylation [14], reductive dehalogenation [15] or reduction of the nitro group [16], (iv) The degradation of ACPs may be initiated with the removal of ammonium ions by the enzyme deaminase followed by the ring cleavage [17] or the dehalogenation [18]. In this section, we describe the bacterial degradation pathways for MCPs, poly-CPs, CNPs, CAPs and CMPs.

### Bacterial degradation of MCPs

MCPs, which are the simplest form of CPs, contain one chlorine atom at the phenolic rings. MCPs include 2-chlorophenol (2CP), 3-chlorophenol (3CP), 4-chlorophenol (4CP), 3-chlorocatechol (3CC), 2-chlorocatechol (2CC) and 4-chlorocatechol (4CC). Chlorocatechols (CCs) were detected as intermediate products of bacterial degradation of MCPs, chlorobenzoates, mono-chlorobiphenyls, 4-chlorosalicylate and 5-chlorosalicylate [19-23]. In this section, we have described the bacterial degradation of 4CP, 2CP and 3CP.

### Bacterial degradation of 4CP

**Figure 2:**
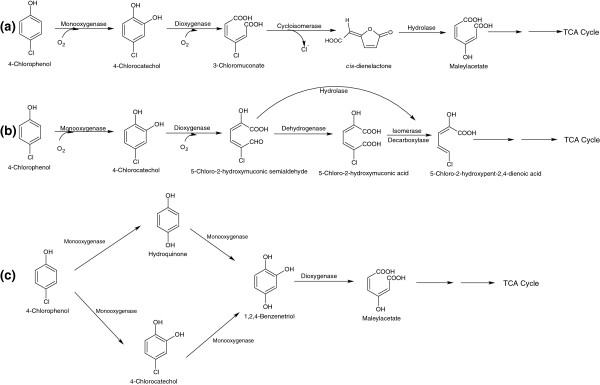
**Bacterial degradation pathways for 4-chlorophenol. (a)** 4-Chlorophenol degradation via modified *ortho*-cleavage, **(b)** 4-chlorophenol degradation via the *meta*-cleavage, **(c)** two pathways of degradation of 4-chlorophenol [4-Chlorocatechol-Benzenetriol pathway (lower) and Hydroquinone pathway (upper)].

Many bacteria that utilize 4CP as their carbon and energy sources have been isolated, including *Pseudomonas knackmussii* B-13 (previously known as *Pseudomonas* sp. B-13) [24,25], *Ralstonia pickettii* LD1 (previously known as *Pseudomonas pickettii* LD1) [26], *Rhodococcus opacus* 1G [27], *Alcaligenes* sp. A7–2 [28], *Alcaligenes xylosoxidans* JH1 [29], *Arthrobacter ureafaciens* CPR706 [30], *Arthrobacter chlorophenolicus* A6 [31], and *Herbaspirillum chlorophenolicum* CPW301 (previously known as *Comamonas testosteroni* CPW301) [32,33]. The bacterial degradation of 4CP occurs via either the CC pathway [34] or the hydroquinone (HQ) pathway [35]. In the CC pathway, 4CP is first converted to 4CC by a 4CP-2-monooxygenase (EC = 1.14.13.-). Further degradation of 4CC then proceeds via the modified *ortho*-ring cleavage or *meta*-ring cleavage pathway [34]. In the modified *ortho*-cleavage pathway, 4CC is cleaved into 3-chloromuconate by a catechol-1,2-dioxygenase (EC 1.13.11.1) [7]. In the second step, 3-chloromuconate is transformed to *cis*-dienelactone through the release of chloride ion by a chloromuconate cycloisomerase (EC 5.5.1.7) [Figure 2a]. In the next step, *cis*-dienelactone is converted to maleylacetate by a dienelactone hydrolase (EC 3.1.1.45) [7]. Maleylacetate is then reduced to 3-oxoadipate by a maleylacetate reductase (EC = 1.3.1.32). In the *meta*-cleavage pathway, 4CC may be cleaved into a toxic compound, 5-chloro-2-hydoxymuconic semialdehyde (5C2HMS) by a catechol-2, 3-dioxygenase (EC = 1.13.11.2) [7,36]. In several cases, 5C2HMS has been identified as a dead end product in the degradation pathway of 4CP [7,36]. However, the complete degradation of 5C2HMS was observed in the 4CP degradation pathway in *Comamonas testosteroni* JH5 [10]. In strain JH4, 5C2HMS was converted to 5-chloro-2-hydroxypenta-2,4-dienoic acid, which was further degraded via intermediates of the TCA cycle [Figure 2b].

In addition to the modified-*ortho* or *meta*-ring cleavage pathway of 4CC, there is another pathway for degradation of 4CC, which is here designated as the 4CC-Benzenetriol (4CC-BT) pathway. In this pathway, 4CC is first hydroxylated to 1,2,4-benzenetriol (BT) through the release of chloride ion [12]. BT is then further degraded via ring cleavage and the formation of maleylacetate [12]. The 4CC-BT pathway was observed in the degradation of 4CP in *A. chlorophenolicus* A6 [12].

The 4CP degradation can also occur through the HQ pathway [12,37]. The first step of the HQ pathway is the formation of HQ through the release of chloride ion from 4CP by a 4CP-4-monooxygenase [Figure 2c]. In the next step, HQ is converted to BT, which is then cleaved into maleylacetate by a BT-dioxygenase [12]. A few bacterial strains degrade 4CP via two pathways. For example, *A. chlorophenolicus* A6 degrades 4CP via the HQ pathway as well as the 4CC-BT pathway [12].

### Bacterial degradation of 2CP and 3CP

**Figure 3:**
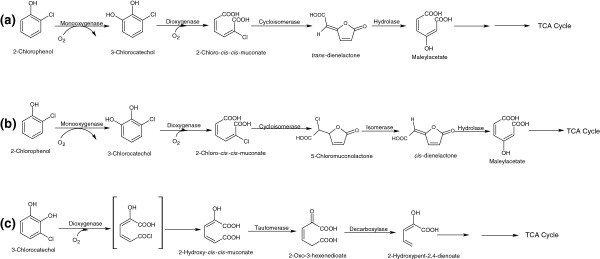
**Bacterial degradation pathways for 2-chlorophenol via 3-chlorocatechol. (a)** Modified *ortho* cleavage pathway, **(b)** new modified *ortho* cleavage pathway, and **(c)***meta-*cleavage pathway of 3-chlorocatechol.

Several 4CP-mineralizing bacteria including *Ralstonia pickettii* LD1 [26], *Rhodococcus opacus* 1G [27] and *Alcaligenes xylosoxidans* JH1 [29] also utilize 2CP and 3CP as their sole carbon and energy sources. Other 2CP-mineralizing bacteria include *Alcaligenes* sp. A7–2 [28] and *Streptomyces rochei* 303 [38]. The bacterial degradation of 2CP occurs via the formation of 3CC, which is further degraded via the modified *ortho*-cleavage pathway or the *meta*-cleavage pathway [7,39]. In the modified *ortho*-cleavage pathway, 3CC is cleaved into 2-chloro-*cis*,*cis*-muconate by a catchol-1,2-dioxygenase [7]. In the next step, a chloromuconate cycloisomerase catalyzes the conversion of 2-chloro-*cis,cis*-muconate to *trans*-dienelactone [7], which degrades further via formation of maleylacetate by dienelactone hydrolase [Figure 3a].

A new modified *ortho*-cleavage pathway of the 3CC was reported in *Rhodococcus opacus* 1CP that degraded 2CP via 3CC [40,41]. The key enzymes of this pathway are chlorocatechol-1,2-dioxygenase, chloromuconate cycloisomerase (CMCI), chloromuconolactone isomerase (CMLI), and dienelactone hydrolase (DELH) [40]. Specifically, chlorocatechol-1,2-dioxygenase catalyzes the conversion of 3CC to 2-chloromuconate, while CMCI converts 2-chloromuconate into 5-chloromuconolactone, CMLI converts 5-chloromuconolactone into *cis*-dienelactone and DELH converts *cis*-dienelactone into maleylacetate, which is further degraded via the TCA cycle [Figure 3b].

In the *meta*-cleavage pathway, there are two possibilities for 3CC degradation: (i) formation of a dead end product [39,42] and (ii) complete mineralization of 3CC [43]. In the first case, a suicide compound, 5-chloroformyl-2-hydroxypenta-2,4-dienoic acid, is formed due to the *meta*-cleavage of 3CC, which inactivates catechol-2,3-dioxygenase (EC = 1.13.11.2), resulting in 3CC accumulation in the media. In the second case, bacteria may utilize 3CC completely. This type of pathway has been observed in the degradation of chloro-aromatics by *Pseudomonas putida* GJ31 [43]. In strain GJ31, 3CC was cleaved into 2-hydroxy-*cis*-*cis*-muconate by a catechol-2,3-dioxygenase that was further degraded completely [43] [Figure 3c]. This pathway has been demonstrated in several other strains that are able to metabolize 3CC, including *Pseudomonas sp.* MG61, *Pseudomonas fluorescens* SK1 and *Pseudomonas veronii* 16-6A [44].

The degradation of 3CP occurred either via the formation of 3CC or via the formation of 4CC that may be further degraded via the modified *ortho*-cleavage pathway or the *meta*-cleavage pathway [7,39].

### Bacterial degradation of poly-CPs

poly-CPs such as dichlorophenols (DCPs), trichlorophenols (TCPs), tetrachlorophenols (TeCPs) and pentachlorophenol (PCP) are more recalcitrant to bacterial degradation than MCPs due to the presence of the two or more chlorine atoms at the phenolic rings. Here, we have described the degradation of 2,4-dichlorophenol (2,4-DCP), 2,4,6-trichlorophenol (2,4,6-TCP), 2,4,5-trichlorophenol (2,4,5-TCP) and PCP.

### Bacterial degradation of 2,4-DCP

**Figure 4:**
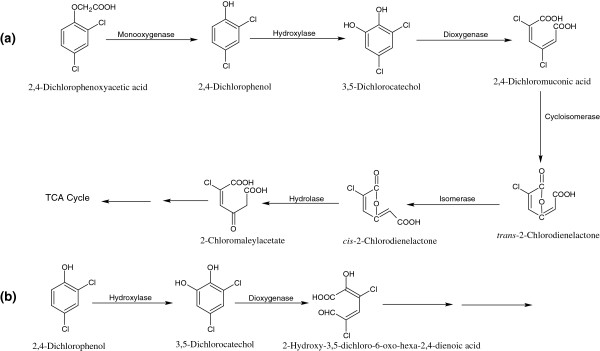
**Bacterial degradation pathways for 2,4-dichlorophenol via *ortho*-cleavage (a) and the distal *meta*-cleavage (b).**

2,4-DCP is the first intermediate in the degradation pathway of a herbicide, 2*,*4*-*dichlorophenoxyacetic acid (2,4-D). Several bacteria that utilize 2,4-DCP as their sole source of carbon and energy have been isolated, including *Pseudomonas* sp. DP-4 [45], *Rhodococcus opacus* 1G [27], *Rhodococcus erythropolis*[46], and *Pseudomonas* sp. NCIB9340 [47]. Wang *et al*. [48] reported the removal of 2,4-DCP by the suspended and immobilized cells of *Bacillus insolitus*. They demonstrated that the immobilized cells showed faster degradation of lower concentrations of 2,4-DCP (10–50 mg/l), whereas the high concentrations (50–200 mg/ml) were removed by immobilized and suspended cells at the same rate [48]. The bacterial degradation of 2,4-D is initiated by the formation of 2,4-DCP by the enzyme, 2,4-dichlorophenoxyacetate-*α*-ketoglutarate dioxygenase (EC = 1.14.11.-) [49]. 2,4-DCP is further degraded via the formation of 3,5-dichlorocatechol by a 2,4-DCP-hydroxylase [EC = 1.14.13.20] [50]. In the third step, 3,5-dichlorocatechol is *ortho*-cleaved to 2,4-dichloromuconic acid by 3,5-dichlorocatechol dioxygenase (EC = 1.13.11.-) (Figure 4a). In the next step, 2,4-dichloromuconic acid isomerase (EC = 5.2.1.10) catalyzes the conversion of 2,4-dichloromuconic acid to *trans*-2-chlorodienelactone via the removal of one chloro group, which is further converted to *cis*-2-chlorodienelactone by an isomerase that is subsequently degraded via formation of chloromaleylacetate by a hydroxylase [50]. The chloromaleylacetate is further degraded to maleylacetate by removal of the chloro group and then to 3-oxodipic acid by a maleylacetate reductase (EC = 1.3.1.32). Koh *et al*. [51] reported that *o*-cresol grown cells of *Cupriavidus necator* JMP222 (a derivative of *C. necator* JMP134 that had lost plasmid pJP4) degraded 2,4-DCP via a distal *meta*-cleavage pathway. In that process, 2,4-DCP is first oxidized to 3,5-dichlorocatechol, which is subsequently degraded via a distal *meta*-cleavage pathway through the formation of 2-hydroxy-3,5-dichloro-6-oxo-hexa-2,4-dienoic acid (Figure 4b).

### Bacterial degradation of 2,4,6-TCP

**Figure 5:**
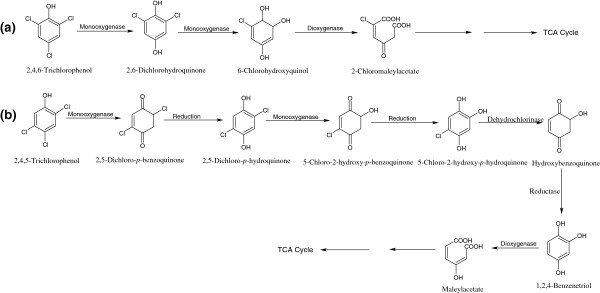
Bacterial degradation pathway for 2,4,6-trichlorophenol (a), and 2,4,5-trichlorophenol (b).

Many bacteria that utilize 2,4,6-TCP as their sole carbon and energy source have been isolated and characterized including *Azotobacter* sp. Gp1 [52], *Ralstonia pickettii*[53], *Cupriavidus necator*[54,55], *Nocardioides* sp. K44 [56] and *Novosphingobium lentum* MT1 [57]. Bacterial degradation of 2,4,6-TCP was well-characterized in *Cupriavidus necator* JMP134 [54,55]. In the initial step of the TCP degradation, a reduced flavin adenine dinucleotide (FADH_2_)-utilizing monooxygenase catalyzes the conversion of 2,4,6-TCP to 6-chlorohydroxyquinol via the formation of 2,6-dichlorohydroquinone [58]. 6-Chlorohydroxyquinol is then further cleaved to 2-chloromaleylacetate by 6-chlorohydroxyquinol-1,2-dioxygenase, which is subsequently converted to maleylacetate by removal of the chloro group (Figure 5a).

### Bacterial degradation of 2,4,5-TCP

*Burkholderia phenoliruptrix* AC1100 (previously known as *B. cepacia*) uses 2,4,5-TCP as the sole source of carbon and energy [59,60]. The first step in degradation of 2,4,5-TCP involves conversion of 2,4,5-TCP to 2,5-dichloro-*p*-benzoquinone (DiCBQ) by FADH_2_-dependent-2,4,5-TCP-4-monooxygenase (TftD) [EC = 1.14.14.-] [60,61]. DiCBQ is then reduced to 2,5-dichloro-*p*-hydroquinone (2,5-DiCHQ) by NADH. In the next step, 2,5-DiCHQ is oxidized to 5-chloro-2-hydroxy-*p*-benzoquinone by TftD, which is then further reduced to 5-chloro-2-hydroxy-*p*-hydroquinone (CHHQ) [60,61]. Another enzyme, flavin reductase TftC, supplies FADH_2_ as a co-substrate to TftD [60,61]. In the next step, dehydrochlorinase TftG catalyzes the conversion of CHHQ to hydroxybenzoquinone, which is reduced to BT by hydroxylbenzoquinone reductase (EC = 1.6.5.7) [62]. BT is subsequently converted to maleylacetate by BT-1,2-dioxygenase (EC = 1.13.11.37) (Figure 5b).

### Bacterial degradation of PCP

**Figure 6:**
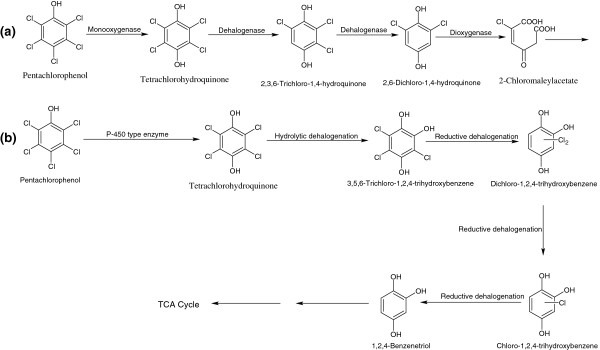
**Bacterial degradation pathways for pentachlorophenol in *Sphingomonas chlorophenolicum *** **L-1 (a), and *Mycobacterium *** **strains (b).**

PCP degradation is initiated by the formation of tetrachlorohydroquinone (TeCHQ) due to hydroxylation at the *para*-position by either PCP-4-monooxygenase (EC = 1.14.13.50) [63-65] or cytochrome P-450 type enzyme [66,67] [Figure 6]. In *Sphingomonas chlorophenolicum* L-1 (previously known as *Sphingomonas chlorophenolicum* ATCC 39723), PCP-4-monooxygenase (PcpA) catalyzes the conversion of PCP to TeCHQ via the removal of chloride ions [63-65]. In the next step, TeCHQ is sequentially dehalogenated to 2,6-dichloro-1,4-hydroquinone (2,6-DCHQ) by a TeCHQ-reductive dehalogenase (EC = 1.8.99.-). The further degradation of 2,6-DCHQ occurs via ring cleavage by the 2,6-DCHQ-1,2-dioxygenase, leading to formation of 2-chloromaleylacetate that is further degraded via the TCA cycle [63-65]. In *Mycobacterium chlorophenolicum* PCP-1 and *Mycobacterium fortuitum* CG-2 (formerly *Rhodoccocus* strains), PCP is hydroxylated to TeCHQ by a membrane bound cytochrome P-450 type enzyme [66,67]. Subsequently, TeCHQ undergoes hydrolytic dehalogenation followed by reductive dehalogenation to form dichloro-1,2,4-trihydroxybenzene, which produces BT after two successive reductive dehalogenation [68].

### Bacterial degradation of CNPs

CNPs are nitro derivatives of MCPs. Examples include 2-chloro-4-nitropheol (2C4NP), 4-chloro-2-nitrophnol (4C2NP), 4-chloro-3-nitrophenol (4C3NP), 2-chloro-5-nitrophenol (2C5NP) and 2-chloro-3-nitrophenol (2C3NP). In this section, we have summarized the bacterial degradation of various CNPs.

**Figure 7:**
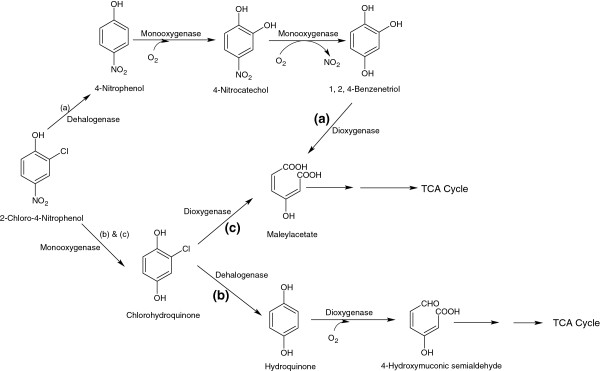
**Bacterial degradation pathways for 2-chloro-4-nitrophenol in (a) *Burkholderia *** **sp. SJ98, (b) *Burkholderia *** **sp. RKJ 800 and *Rhodococcus imtechensis *** **RKJ300, and (c) *Arthrobacter nitrophenolicus *** **SJCon.**

The degradation of 2C4NP has been studied in *Burkholderia* sp. SJ98 [15], *Burkholderia* sp. RKJ 800 [14], *Arthrobacter nitrophenolicus* SJCon [69] and *Rhodococcus imtechensis* RKJ300 [70]. Pandey *et al*. [15] proposed a degradation pathway of 2C4NP in *Burkholderia* sp. SJ98 that utilized 2C4NP as the sole carbon, nitrogen and energy sources. The first step of the 2C4NP degradation in strain SJ98 involves the reductive dehalogenation of 2C4NP by 2C4NP-dehalogenase that leads to the formation of 4-nitrophenol (4NP) [Figure 7a]. In the next step, 4NP is converted to 4-nitrocatechol and then to BT, which is further cleaved into maleylacetate by BT-1,2-dioxygenase. Maleylacetate is further degraded via the *β*-ketoadipic acid cycle [15]. Another pathway of the 2C4NP degradation was investigated in *Rhodococcus imtechensis* RKJ 300 [70] and *Bukholderia* sp. RKJ 800 [14]. In this pathway, 2C4NP is first transformed to chlorohydroquinone (CHQ) by 2C4NP-monooxygenase. CHQ is then dehalogenated to HQ by CHQ-dehalogenase [14]. In the next step, HQ is cleaved to *γ*-hydroxymuconic semialdehyde by HQ-1,2-dioxygenase (EC = 1.13.11.66) [Figure 7b]. Arora and Jain [69] reported a new degradation pathway of 2C4NP in *Arthrobacter nitrophenolius* sp. SJCon. In strain SJCon, 2C4NP is first converted to CHQ and then further cleaved to maleylacetate by CHQ-dioxygenase [Figure 7c].

**Figure 8:**
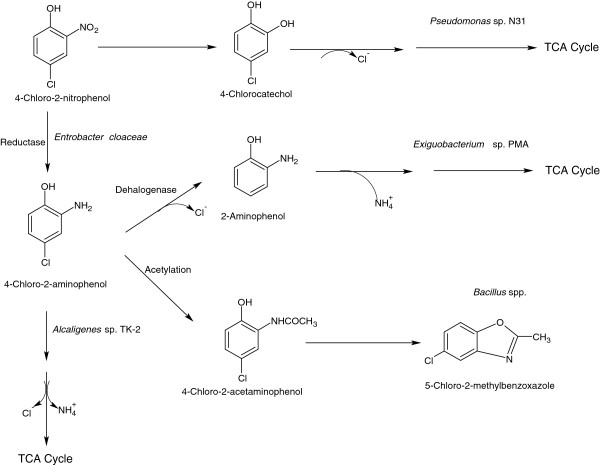
Bacterial degradation pathways for 4-chloro-2-nitrophenol.

The first report of the 4C2NP degradation was documented in 1988 with construction of a genetically engineered bacterium, *Pseudomonas* sp. N31, which utilizes 4C2NP as a sole carbon, nitrogen and energy source [71]. The constructed strain degrades 4C2NP via the formation of 4CC and the release of chloride and nitrite ions [Figure 8]. Beunink and Rehm [72] reported 4C2NP degradation via the formation of 4-chloro-2-aminophenol (4C2AP) by a co-culture of *Enterobacter cloaceae* and *Alcaligenes* sp. TK-2 [Figure 8]. A detoxification mechanism for 4C2NP transformation has been proposed for two *Bacillus* species [73,74]. In this mechanism, detoxification is initiated by the formation of 4C2AP, which acetylates into 4-chloro-2-acetaminophenol (4C2AAP). 4C2AAP is then converted to a non-toxic compound, 5-chloro-2-methylbenzoxazole [Figure 8]. Another investigation of complete mineralization of 4C2NP was published following the isolation of a 4C2NP-mineralization bacterium, *Exiguobacterium* sp. PMA [16]. This strain initiates 4C2NP degradation by the formation of 4C2AP via a reduction mechanism, which is further dehalogenated into 2-aminophenol (2AP) through the release of chloride ions [16]. The further degradation of 2AP proceeds via ring cleavage and the removal of ammonium ions [Figure 8].

**Figure 9:**
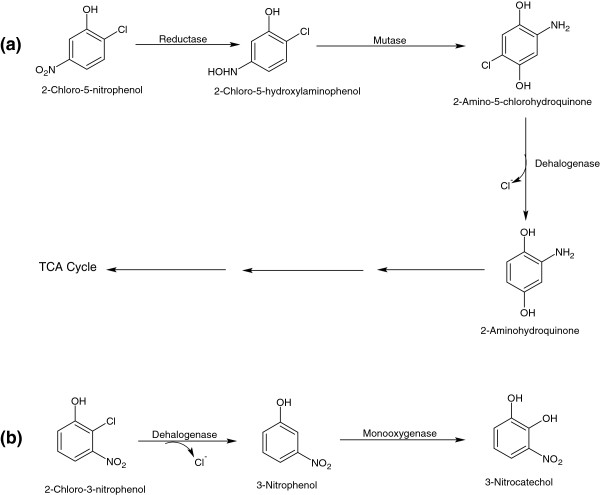
Bacterial degradation pathway for 2-chloro-5-nitrophenol (a), and 2-chloro-3-nitrophenol (b).

The metabolic pathway of 2C5NP has also been studied in *C. necator* JMP134, which utilizes 2C5NP as its sole carbon, nitrogen and energy source [75]. The first step of 2C5NP degradation involves the reduction of 2C5NP to 2-chloro-5-hydroxylaminophenol (2C5HAP) by 3NP-reductase [75]. In the second step, 2C5HAP undergoes Bamberger rearrangement to form 2-amino-5-chlorohydroquinone (2A5CHQ) by mutase [Figure 9a]. In the next step, 2A5CHQ is reductively dehalogenated to 2-aminohydroquinone, which is further degraded by ring cleavage and ammonia release [75].

Pandey *et al*. [76] reported the biotransformation of 2C3NP to 3-nitrocatechol (3NC) in *Burkholderia* sp. SJ98. Initially, 2C3NP is reductively dehalogenated to 3NP, which is further hydroxylated to 3NC [Figure 9b].

### Bacterial degradation of CAPs and CMPs

**Figure 10:**
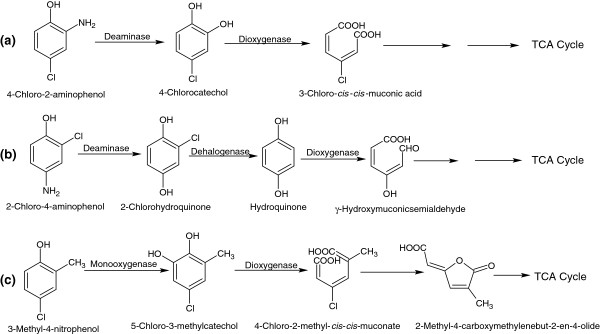
Bacterial degradation pathways for 4-chloro-2-aminophenol (a), 2-chloro-4-aminophenol (b), and 4-chloro-2-methylphenol (c).

CAPs are amino derivatives of MCPs that are used in the manufacture of dyes. Examples include 4-chloro-2-aminophenol (4C2AP) and 2-chloro-4-aminophenol (2C4AP). Bacterial degradation of 4C2AP was studied in the Gram negative bacterium, *Burkholderia* sp. RKJ 800, which utilizes 4C2AP as a sole carbon and energy source [17]. The degradation of 4C2AP is initiated by the release of ammonium ion and the formation of 4CC by a deaminase. In the next step, 4CC is cleaved to *cis*, *cis*-chloromuconic acid by 4CC-1,2-dioxygenase (Figure 10a). Conversely, the bacterial degradation of 2C4AP was studied in a Gram positive bacterium, *Arthrobacter* sp. SPG, which utilized 2C4AP as its sole source of carbon and energy [18]. The first step of 2C4AP degradation involves removal of the ammonium ion by deaminase, which leads to formation of CHQ that is then dehalogenated to HQ by a CHQ-dehalogenase (Figure 10b). In the next step, HQ is cleaved to *γ*-hydroxymuconic semialdehyde by HQ-1,2-dioxygenase (EC = 1.13.11.66) [18].

CMPs are methyl derivatives of CPs used for the manufacture of herbicides such as 4-chloro-3-methylphenol and 4-chloro-2-methylphenol. Lechner *et al*. [77] investigated the degradation pathway of 4C2MP in a Gram negative strain, S-1. 4C2MP is first converted to 5-chloro-3-methylcatechol, which is *ortho*-cleaved into 4-chloro-2-methyl-*cis*-*cis*-muconate and then further degraded via the formation of 2-methyl-4-carboxymethylenebut-2-en-4-olide (Figure 10c).

### Anaerobic degradation of CPs

**Figure 11:**
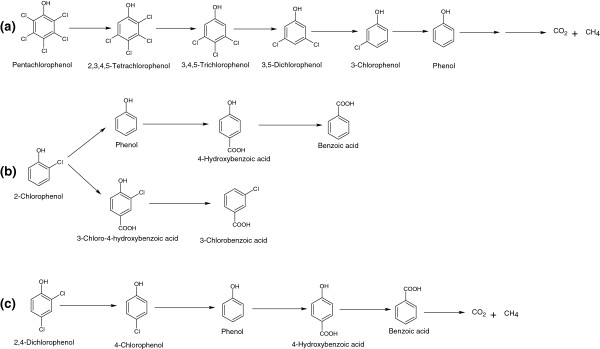
**Anaerobic degradation of CPs and poly-CPs. (a)** Anaerobic dehalogenation and degradation of pentachlorophenol, **(b)** Two biotransformation pathways for 2-chlorophenol, and **(c)** Anaerobic mineralization for 2,4-dichlorophenol.

Anaerobic degradation of CPs is well studied in bacteria or various enrichment cultures derived from sediments collected from a variety of the sources [8]. Anaerobic degradation of various CPs proceeds via reductive dehalogenation in which chlorine atoms are replaced by hydrogen atoms [8]. In fact, the reductive dehalogenation is a crucial step for the anaerobic biodegradation of CPs especially for poly-CPs. Several poly-CPs are recalcitrant towards aerobic bacterial attack and can be reductively dehalogenated into lesser chlorinated phenols that further mineralized easily. PCP may be reductively dehalogenated to 2,3,4,5-tetrachlorophenol (2,3,4,5-TeCP), then to 3,4,5-trichlorophenol (3,4,5-TCP), then to 3,5-dichlorophenol, then to 3CP and finally to phenol which further degraded to CH_4_ and CO_2_ by anaerobic bacteria [78,79] (Figure 11a). The combination of phenol-dehalogenating and phenol-degrading cultures was used for complete mineralization of PCP under anaerobic conditions [80]. In this process, a phenol-dehalogenating culture dehalogenates PCP to phenol under anaerobic conditions. The phenol is then further degraded by phenol-degrading culture under iron reducing or sulfate reducing conditions [80]. Becker *et al*. [81] studied two biotransformation pathways for 2CP in the anaerobic sediment slurry reactors. In the first pathway, 2CP is reductively dehalogenated to phenol, then carboxylated to 4-hydroxybenzoate and finally dehydroxylated to benzoate (Figure 11b). In the second pathway, 2CP is *para*-carboxylated to 3-chloro-4-hydroxybenzoate, which is further dehydroxylated to 3-chlorobenzoate.The mineralization of ^14^C-radiolabeled 4CP, 2CP, and 2,4-DCP to ^14^CH_4_ and ^14^CO_2_ was studied in acclimated sludge [82]. In this process, 4CP is mineralized via phenol, 4-hydroxybenzoate and benzoate, while 2,4-DCP is mineralized via 4CP, phenol, 4-hydroxybenzoate and benzoate [82] (Figure 11c).

Methanogenic, sulfate reducing, iron-reducing and denitrifying conditions favor anaerobic degradation and reductive dechlorination of CPs. Anaerobic degradation and dechlorination of CPs have been extensively studied under methanogenic conditions [78,83-87]. A PCP acclimated methanogenic consortium reductively dechlorinated PCP and TeCPs [83]. In this process, PCP is first dechlorinated to 2,3,4,5-TeCP, 2,3,4,6-tetrachlorophenol and 2,3,5,6-tetrachlorophenol. These TeCPs are then further dehalogenated to TCPs, DCPs and MCPs. Another methanogenic enrichment culture derived from sewage sludge transformed 2,4,6-TCP, 2,4,5-TCP and 3,4,5-TCP [84]. In this process, 2,4,6-TCP is reductively dechlorinated to 4CP via 2,4-DCP, whereas 2,4,5-TCP and 3,4,5-TCP are dehalogenated to 3CP via 3,4-DCP [84]. The reductive dechlorination of 12 isomers of CPs and poly-CPs including MCPs, DCPs, TCPs, TeCPs and PCP was also investigated using methanogenic cultures [85]. Takeuchi *et al*. [86] reported dehalogenation and transformation of 19 isomers of CPs under methanogenic conditions. A fresh water sediment mineralized 2,4-DCP into CO_2_ and methane via 4CP, phenol and benzoate [87].

The mineralization of CPs has been found to be coupled with sulfate reduction. Haggblom and Young [88] developed a CPs-mineralizing sulfate reducing consortia from estuarine sediment that was maintained on 2CP, 3CP or 4CP as the only source of carbon and energy for several years [89]. Their experiments utilizing a 4CP-utilizing consortium revealed that mineralization of 4CP into CO_2_ was coupled to sulfate reduction, and that 4CP depletion did not occur in the absence of sulfate. In this reaction, sulfate, thiosulfate or sulfite were used as electron acceptors [89]. The coupling of sulfate reduction with mineralization of CPs was also observed in degradation of 2CP or 4CP by sulfate reducing enrichment cultures derived from Hudson River sediment [90].

Under denitrifying conditions, the 2CP degradation was studied in enrichment cultures derived from activated sludge samples [91]. The presence of nitrate was essential as electron acceptors for the mineralization of 2CP into CO_2_[91]. Sanford and Tiedje [92] studied dechlorination and subsequent degradation of MCPs and DCPs in anaerobic microcosms supplemented with 1 mM or 5 mM nitrate.

CPs degradation is associated with reduction of Fe^3+^ to Fe^2+^. An anaerobic enrichment culture derived from Hudson River sediments mineralized 2CP, 3CP and 4CP with concomitant reduction of Fe^3+^ to Fe^2+^[93]. Several factors may affect dechlorination of CPs and reduction of Fe. For example, a low amount of nitrate enhances reductive dechlorination of PCP and Fe(III) reduction, while high concentrations of nitrate inhibit reductive dechlorination and Fe(III) reduction [94].

The reductive dehalogenation of MCPs and DCPs was investigated in the anaerobic sediment samples of estuarine Lake Shinji and Lake Nakaum [95]. Estuarine sediment enrichment cultures of lake Shinji dehalogenated 2CP, 3CP and 2,6-DCP, whereas enrichment cultures of Lake Nakaum dehalogenated 3CP and 2,6-DCP [95]. The dehalogenated product of MCPs was phenol, which was further degraded by the formation of benzoic acid. Itoh *et al*. [96] identified the bacterial consortia involved in dehalogenation of MCP into phenol and transformation of phenol to benzoic acid using polymerase chain reaction-denaturing gradient gel electrophoresis (PCR-DGGE) of the 16S rRNA gene in the enrichment sample of Lake Shinji. The 4CP-dechlorinating culture had two dominant bacteria, in which one belonged to *Dehalobacter* sp. In the phenol transforming culture, *Cryptanaerobacter phenolicass* was present.

Li *et al*. [97] established a simple anaerobic upflow column system (15 cm long, 5 cm inner diameter) for complete PCP-mineralization using a microbial consortium requiring only lactate as an external nutrient. Anaerobic microbes dehalogenated PCP to 3CP and phenol using external lactate as an electron donor [97]. The further degradation of 3CP and phenol proceeded without an external electron donor and the nitrogen required for degradation was supplied by nitrogen-fixation [97]. The potential dechlorinators, *Dehalobacter* and *Desulfitobacterium*, and the phenol/3CP fermentative or syntrophic degraders, *Cryptanaerobacter* and *Syntrophus*, were found at the bottom of the column, whereas the nitrogen-fixing facultative anaerobe, *Rhizobiales*, was detected in the top of the upflow column, and other possible nitrogen-fixers were found at both the bottom and top of the upflow column [97].

A variety of pure bacterial cultures have been characterized for their ability to dechlorinate CPs under anaerobic conditions [98-101]. For example, several species of *Desulfitobacterium* with dehalogenating capabilities toward various CPs have been isolated and characterized, including *Desulfitobacterium hafniense* PCP-1 [98], *D. hafniense* DCB-2 [99], *D. dehalogenans* IW/IU-DC1 [102] and *D. chlororespirans*[103]. These strains utilize CPs as electron acceptors for growth during the oxidation of electron donating chemicals in a process known as halorespiration [8]. Villemur [98] reported that *D. hafniense* PCP-1 isolated from a methanogenic consortium was able to dehalogenate PCP to 3CP via the formation of 3,4,5-TCP and 3,5-DCP. Strain PCP-1 was also capable of dehalogenation of other TCPs and DCPs, but unable to dehalogenate MCPs [98]. *D. hafniense* strain DCB-2 removed the *ortho*-substituted chorine from 2,4,6-TCP, 2,4,5-TCP, PCP, 2,4-DCP, and *meta*-substituted chlorine from 3,5-DCP [98]. Apart from *Desulfitobacterium* spp., other dehalorespirating bacteria include *Desulfomonile tiedje* DCB-1 [100] and *Anaeromyxobacter dehalogenans*[101]. Mohn and Kennedy [100] reported the dehalogenation of PCP into 2,4,6-TCP by 3-chlorobenzoate-induced cells of a sulfate reducing bacterium, *Desulfomonile tiedje* DCB-1. He and Sandford [104] reported *ortho*-dehalogenation of 2,6-DCP and 2CP to phenol by a facultative anaerobic bacterium, *Anaeromyxobacter dehalogenans* 2CP-C. Recently, Wang *et al.*[105] reported the removal of the *ortho*-chlorines from 2,4,6-TCP by *Dehalobacter* sp. PCP-1 that converted 2,4,6-TCP to 4CP via 2,4-DCP.

### Genetics of bacterial degradation of CPs

The genes responsible for degradation of CPs are located on either plasmids or chromosomal DNA. Plasmid encoded genes include (i) two *tdf* operons on the *C. nector* JMP134 plasmid pJP4 [106], (ii) *tcpRXABCYD* cluster on *C. necator* JMP134 (pJP4) [107], (ii) the *clc* operon on the *Pseudomonas knackmussii* plasmid pB13 (pWR1) [108], and (iv) the *tcb* operon on the *Pseudomonas* sp*.* P51 plasmid pP51 [109]. Two *tdf* gene clusters (*tfdC*_*I*_*D*_*I*_*E*_*I*_*F*_*I*_ and *tfdD*_*II*_*C*_*II*_*E*_*II*_*F*_*II*_) identified on plasmid pJP4 of strain JMP134 encode enzymes for 4CC metabolism. The genes *tfd*C, *tfd*D, *tfd*E and *tfd*F encode the enzymes chlorocatechol-1,2-dioxygenase (EC 1.13.11.-) (TfdC), chloromuconate cycloisomerase (TfdD) (EC = 5.5.1.7), dienelactone hydrolase (TfdE) (EC = 3.1.1.45), and maleylacetate reductase (TfdF) (EC = 1.3.1.32), respectively [106]. Van der Meer *et al*. [109] reported that genes *tcb*C, *tcb*D and *tcb*E located on the operon *tcb* (pP51) encoded a catechol 1,2-dioxygenase II (EC 1.13.11.1), a cycloisomerase II (EC = 5.5.1.7), and a hydrolase II (EC = 3.1.1.45), respectively which degraded 3,4-dichlorocatechol and 3,4,6-trichlorocatechol to chloromaleylacetate. The *clc* operon contains the genes for utilization of CCs on the *Pseudomonas* sp*.* P51 plasmid pP51 [108]. These genes include *cicA*, the gene encoding catechol oxygenase II (EC 1.13.11.1), *clc*B, the gene encoding muconate cycloisomerase II (EC = 5.5.1.7), and *clcD*, the gene encoding dienelactone hydrolase (EC = 3.1.1.45). The genes for degradation of 2,4,6-TCP are located on the *tcpRXABCYD* cluster from *C. necator* JMP134 (pJP4) [107]. The gene *tcp*A encodes a reduced flavin adenine dinucleotide (FADH2)-dependent monooxygenase (TcpA) (EC = 1.14.13-) that converts 2,4,6-TCP to 6-chlorohydroxyquinol. TcpA needs FADH2 that is supplied by the putative flavin reductase (TcpX) encoded by the *tcpX* gene [107]. The *tcpB* gene may also encode flavin reductase activity because it showed sequence similarity to genes coding for nitroreductases [107]. The gene *tcp*C encodes an enzyme 6-chlorohydroxyquinol-1,2-dioxygenase (TcpC) (EC = 1.13.11.-) that cleaves chlorohydoxyquinol to 2-chloromaleylacetate. The gene *tcp*D encodes an enzyme maleylacetate reductase (TcpD) (EC = 1.3.1.32) that converts chloromaleylacetate to *β*-ketoadipate. The gene *tcpR* is a regulator that controls the expression of all *tcp* genes whereas the function of *tcp*Y is not clear [107]. In *Ralstonia picketti* DTP0602, two gene clusters (*hadXABC* and *hadYD*) are involved in the conversion of 2,4,6-TCP to 3-oxoadipate, where *hadXABC* and *hadYD* are regulated by *hadR* and *hadS*, respectively [110]. Torii *et al*. [111] investigated how HadR regulates 2,4,6-TCP catabolic pathway gene expression in *Ralstonia pickettii* DTP0602. They found that purified HadR binds to the *hadX* promoter and HadR–DNA complex formation is induced in the presence of 16 types of substituted phenols, including CPs, nitrophenols and tribromophenols.

A gene cluster containing four genes (*clcA2, clcB2, clcD2* and *clcF*) involved in a new modified *ortho*-cleavage pathway of 3CC was identified in *Rhodococcus opacus* 1CP [47]. The genes *clc*A2, *clc*B2, *clc*D2 and *clc*F encode the enzymes 3-chlorocatechol-1,2-dioxygenase (ClcA2), chloromuconate cycloisomerase (ClcB2), dienelactone hydrolase (ClcD2) and muconolactone isomerase-related enzyme (ClcF), respectively. This organism also contains a second cluster of chlorocatechol degradation genes that are similar to the proteobacterial genes [41].

A 4CP-degradation gene cluster (*cph* genes) was identified in *A. chlorophenolicus*[12]. This gene cluster contains 10 open reading frames that show similarity to the genes encoding the enzymes involved in CP degradation. Several open reading frames encode enzymes with similar functions. For example, two genes, *cphA*-1 and *cph*-11, encode functional hydroxyquinol-1,2-dioxygenase. A mutant strain constructed by disturbing the gene *cphA*-1 by site-directed mutagenesis was unable to utilize 4CP as the sole source of carbon energy. Other genes present on this cluster include *cph*C-I*, cphC*-II, *cphF*-I*, cphF*-II, *Cph* B, *Cph*X, *CphR* and *Cph*S. The genes *cphC*-I and *cph*C-II encode putative monooxygenase, whereas *cph*F-1 and *cph*F-11 encode putative maleylacetate reductase and *cph*B encodes a NADH:flavin adenine dinucleotide oxidoreductase. The roles of the remaining genes in the *cph* gene cluster have yet to be determined [112]*.*

The *ccaBARCD* gene cluster is involved in CC degradation in *Pseudomonas reinekei* MT1 [112]. The genes *ccaA, ccaB, ccaC* and *ccaD* encode the enzymes catechol-1,2-dioxygenase, (chloro) muconate cycloisomerase, *trans*-dienelactone hydrolase and maleylacetate reductase, respectively. The gene, *cca*R is a putative regulator homologous to regulators of the IclR-type family [112].

Genes for degradation of 2,4,5-TCP have been identified and characterized from *Burkholderia phenoliruptrix* AC1100 [61,62,113-115]. Two gene clusters, *tftCD*, and *tftEFGH*, are involved in conversion of 2,4,5-TCP to 3-oxoadipate in *B. phenoliruptrix* AC1100 [61,62,113-115].

Several genes (*pcpB, pcpC*, *pcpA* and *pcp E)* involved in PCP degradation have been identified and characterized from *Sphingomonas chlorophenolicum* L-1 [116]. The genes *pcpB, pcpC, pcpA* and *pcpE* encode the enzymes PCP-4-monooxygenase (PcpB) (EC = 1.14.13.50), TeCH-reductive dehalogenase (PcpC) (EC = 1.8.99-), DiCHQ-1,2-dioxygenase (PcpA) (1.13.11.-), maleylacetate reductase (EC = 1.3.1.32), respectively. PcpB is a flavin monooxygenase that converts PCP to TeCHQ via hydroxylation at the *para*-position with removal of the chloride ion in the first step of the bacterial degradation of PCP. PcpB has broad substrate specificity and catalyzes reaction of various substituted aromatic compounds [117]. The *pcp*B gene has also been detected in three other strains of *Sphingonium chlorophenolicum* (RP-2, SR-3 and ATCC 33790). An identical *pcp*B gene sequence was found in three strains (L-1, RP-2, SR-3) [118,119], whereas the *pcp*B gene sequence of *Sphinogomonads strain* UG-30 showed 90% sequence similarity with that of *Sphingonium chlorophenolicum* ATCC 39723 [120-122]. Homologues of the *pcp*B gene have also been detected in the polychlorinated degrading bacterium, *Novosphingonium* sp. strain MT1 [123], and in two non-PCP degrading β- and γ- proteobacterial strains [124]. In the second step of the PCP degradation, PcpC catalyzes the reductive dehalogenation of TeCHQ to 2,6-DCHQ, which is further cleaved to 2-chloromaleylacetate by PcpA. PcpE converts 2-chloromaleylacetate to 3-oxoadipate via maleylacetate. Another gene, *pcp*R is a LysR-type regulator that is essential to the induction of *pcpB*, *pcpA*, and *pcpE.*

### Genetics of reductive dehalogenation

Reductive dehalogenation of CPs and poly-CPs is generally carried out by chlorophenol reductive dehalogenases (CprA) encoded by the *cprA* gene, which have been well-studied in *Desulfitobacterium hafniense* PCP-1, *D. dehalognase* IW/IU-DC1, and *D. chlororespirans*[98]. The *cpr*A genes are associated with *cpr* gene clusters that also encode several accessory proteins (e.g., CprA-anchor protein [98,125], chaperones, regulators [126]). The *cpr* gene clusters composed of eight genes (*cprT*, *cprK*, *cprZ*, *cprE*, *cprB*, *cprA*, *cprC*, and *cprD*) have been identified in the genome of *Desulfitobacterium dehalogenans* IW/IU-DC1 and *Desulfitobacterium hafniense* DCB-2 [126,127]. CprK, a member of the CRP-FNR (cAMP-binding protein/fumarate nitrate reduction regulatory protein) family regulators, control transcription of the *cpr* genes [128]. The mechanism responsible for regulation of transcription of *cpr* genes has been investigated [128]. An effector domain of CprK interacts with a chlorinated aromatic compound with high affinity which induces its binding to an upstream target DNA sequence known as the “dehalobox to activate the transcriptions of the *cpr* genes [128].

Four genes (*cprA2*, *cprA3*, *cprA4* and *cprA5*) encoding the putative chloroaromatic reductive dehalogenases (CprA2-A5) have been identified in *D. hafniense* PCP-1 [129-132]. Two gene products (CprA3 and CprA5) have been purified and characterized. CprA3 catalyzes *ortho*-dechlorination of highly chlorinated phenols including PCP, 2,3,4,5-TeCP, 2,3,4-TCP, 2,4,6-TCP and 2,3,6-TCP, whereas CprA5 catalyzes *meta*-dechlorination of 3,5-DCP and 2,3,5-TCP, *para*-dechlorination of PCP, 2,3,4,5-TeCP and 3,4,5-TCP, and *ortho*-dechlorination of 2,4,6-TCP, 2,4,5-TCP and 2,4-DCP [131,132]. The dehalogenation activities of the products of another two genes (*cpr*A2 and *cpr*A3) are not yet known. The transcription levels of the *cpr*A2, *cpr*A3, *cpr*A4 and *cpr*A5 genes were measured in strain PCP cultures exposed to CPs by reverse transcription-quantitative PCR [133]. The genes *cpr*A2 and *cpr*A3 were upregulated in cultures amended with 2,4,6-TCP, whereas only *cpr*A5 was upregulated in 3,5-DCP-amended cultures. In PCP-amended cultures grown for 12 h, *cp*rA2 and *cpr*A3 were upregulated, but *cpr*A5 was not. The gene, *cpr*A4 was not upregulated significantly in cultures containing any tested CPs [133].

A non-CprA reductive dehalogenase known as CrdA from *D. hafniense* strain PCP-1 cultures amended with 2,4,6 TCP has been isolated and characterized. CrdA catalyzes *ortho*-dehalogenation of PCP and 2,4,6-TCP [134]. The gene (*crd*A) encoding CrdA has been cloned and sequenced from strain PCP-1 and also detected in several other strains of *Desulfitobacterium*[134]. Gauthier *et al*. [129] monitored the expression of the *crd* gene in *Desulfitobacterium* strains and transcripts of *crd*A were detected in *D. hafniense* strains PCP-1, DCB-2 and TCE-1.

## Conclusions

The bacterial degradation of MCPs and poly-CPs has been extensively studied and several pathways have been proposed for degradation of MCPs and poly-CPs. The bacterial degradation of CPs and poly-CPs proceeded via formation of the corresponding CCs or the corresponding (chloro)HQs. The genes involved in the degradation of MCPs and poly-CPs have also been identified and characterized from CPs-degrading bacteria.

CAPs and CMPs are highly toxic compounds, and few studies have been conducted to investigate the biodegradation of these compounds. More CAPs and CMPs-degrading bacteria must be isolated to investigate the genetic and biochemical mechanism by which these compounds are degraded.

Anaerobic degradation of CPs has also been studied, and it has been established that MCPs and poly-CPs are initially dehalogenated to phenol, which is further transformed to benzoic acid and then mineralized to CO_2_ under anaerobic conditions. However, further study is needed to elucidate the genetic and enzymatic basis of this mechanism. Furthermore, anaerobic degradation of other CPs such as CNPs, CAPs and CMPs should also be studied.

## Abbreviations

CPs: Chlorophenols; MCPs: Monochlorophenols; poly-CPs: Polychlorophenols; CNPs: Chloronitrophenols; CAPs: Chloroaminophenols; CMPs: Chloromethylphenols; 2CP: 2-Chlorophenol; 3CP: 3-Chlorophenol; 4CP: 4-Chlorophenol; CC: Chlorocatechol; 4CC: 4-Chlorocatechol; 3CC: 3-Chlorocatchol; 2CC: 2-Chlorocatechol; HQ: Hydroquinone; 5C2HMS: 5-Chloro-2-hydroxymuconic semialdehyde; BT: 1,2,4-Benzenetriol; 4CC-BT pathway: 4-Chlorocatechol-benzenetriol pathway; CMCI: Chloromuconate cycloisomerase; CMLI: Chloromuconolactone isomerase; DELH: Dienelactone hydrolase; DCPs: Dichlorophenols; TCPs: Trichlorophenols; TeCPs: Tetrachlorophenols; PCP: Pentachlorophenol; 2,4-DCP: 2,4-Dichlorophenol; 2,4,6-TCP: 2,4,6-Trichlorophenol; 2,4,5-TCP: 2,4,5-Trichlorophenol; 2,4-D: 2*,*4*-*Dichlorophenoxyacetic acid; DiCBQ: 2,5-Dichloro-*p*-benzoquinone; 2,5-DiCHQ: 2,5-Dichlorohydroquinone; CHHQ: 5-Chloro-2-hydroxy-*p*-hydroquinone; TeCHQ: Tetrachlorohydroquinone; 2,6-DCHQ: 2,6-Dichloro-1,4-hydroquinone; 2C4NP: 2-Chloro-4-nitropheol; 4C2NP: 4-Chloro-2-nitrophnol; 4C3NP: 4-Chloro-3-nitrophenol; 2C5NP: 2-Chloro-5-nitrophenol; 2C3NP: 2-Chloro-3-nitrophenol; 4NP: 4-Nitrophenol; CHQ: Chlorohydroquinone; 4C2AP: 4-Chloro-2-aminophenol; 4C2AAP: 4-Chloro-2-acetaminophenol; 2AP: 2-Aminophenol; 2C5HAP: 2-Chloro-5-hydroxylaminophenol; 2A5CHQ: 2-Amino-5-chlorohydroquinone; 3NC: 3-Nitrocatechol; 4C2AP: 4-Chloro-2-aminophenol; 2C4AP: 2-Chloro-4-aminophenol; 2,3,4,5-TeCP: 2,3,4,5-Tetrachlorophenol; 3,4,5-TCP: 3,4,5-Trichlorophenol; TCA Cycle: Tricarboxylic acid cycle.

## Competing interests

The authors declare that they have no competing interests.

## Authors’ contributions

PKA collected all the relevant publications, arranged the general structure of the review, drafted the text and produced figures. HHB revised and formatted the review and also help to draft the manuscript. All authors read and approved the final manuscript.
